# In-Depth Experimental Analysis of Influence of Electroplated Gold Thickness on Thermal and Electro-Optical Properties of mid-IR AlInAs/InGaAs/InP Quantum Cascade Lasers

**DOI:** 10.3390/ma14237352

**Published:** 2021-11-30

**Authors:** Dorota Pierścińska, Kamil Pierściński, Grzegorz Sobczak, Katarzyna Krajewska, Krzysztof Chmielewski, Aleksandr Kuźmicz, Krzysztof Piskorski, Piotr Gutowski

**Affiliations:** Łukasiewicz Instytut Mikroelektroniki i Fotoniki (Ł-IMiF), 02-668 Warsaw, Poland; kamil.pierscinski@imif.lukasiewicz.gov.pl (K.P.); grzegorz.sobczak@imif.lukasiewicz.gov.pl (G.S.); katarzyna.krajewska@imif.lukasiewicz.gov.pl (K.K.); krzysztof.chmielewski@imif.lukasiewicz.gov.pl (K.C.); aleksandr.kuzmicz@imif.lukasiewicz.gov.pl (A.K.); krzysztof.piskorski@imif.lukasiewicz.gov.pl (K.P.); piotr.gutowski@imif.lukasiewicz.gov.pl (P.G.)

**Keywords:** quantum cascade lasers, thermoreflectance, Raman spectroscopy, gold electroplating

## Abstract

In this paper, we have examined the influence of electroplated gold thickness on the thermal and electro-optical properties of mid-IR AlInAs/InGaAs, InP QCLs. The experimental results show a significant reduction of the temperature of QCL active region (AR) with increasing gold layer thickness. For QCLs with 5.0 μm gold thickness, we observed a 50% reduction of the active region temperature. An improvement of key electro-optical parameters, that is, threshold current density and maximum emitted power for structures with thick gold, was observed. The results of micro-Raman characterization show that the electroplated gold layer introduces only moderate compressive strain in top InP cladding, which is well below the critical value for the creation of misfit dislocations.

## 1. Introduction

Quantum cascade lasers (QCLs) have become the laser sources of choice in the mid-infrared. Owing to their range of available wavelengths [1], wide tunability [2], single mode emission [3] and high optical power at room temperature [4], quantum cascade lasers can provide efficient and versatile single mode light sources for molecular spectroscopy of trace gases and air pollutants. QCLs are also a promising source for mid-IR free-space communications at multi-gigabit transmission rates [5,6]. The specific characteristic of QCLs is that they operate at a relatively high current and voltage. Due to relatively low electrical energy conversion efficiency, most of the supplied electric power is converted to heat. This process is especially important because, even for the best performing devices, the wall-plug efficiency of mid-IR QCLs operating at room temperature is still limited to about 20% under pulse-mode operation [7]. The excess Joule heating localized mostly inside the active region [8] raises the device’s internal temperature [9], resulting in a decrease of output power and device degradation [10]. The heat dissipation plays a critical role in the device performance of mid-IR QCLs. Among the methods used to reduce the laser overheating are the optimization of the laser active region and waveguide design, the processing technology, mounting and device packaging [11,12,13,14,15].

In this paper, we analyze the influence of electroplated gold layer thickness on the performance of ridge waveguide (RW) AlInAs/InGaAs/InP QCLs. In the case of investigated devices (RW QCLs mounted epi-side up), the thick gold layer has a significant effect on the thermal behavior of the laser as the thermal conductivity of gold is high (315 W/mK [16]) and it is located close to the heat source (active area of the laser). The AlInAs/InGaAs/InP QCLs with different thicknesses of gold layer have been fabricated and characterized thermally, showing a significant reduction of temperatures in AR (active region) and an improvement of key electro-optical parameters for structures with 5.0 μm gold layer thickness. We have examined key electro-optical, thermal and spectral parameters of the investigated devices. The processing-induced strain in devices with different gold layer thicknesses has been analyzed by micro-Raman spectroscopy. The results clearly confirm that a thick electroplated gold layer on top of the contact layer can be as efficient in heat extraction from the active region as a more complicated buried heterostructure approach.

## 2. Materials

The examined QCLs are based on the strain-compensated In_0_._67_Ga_0_._33_As/In_0_._36_Al_0_._64_As active region, designed for emission at the wavelength around 4.5 μm. The active region design is based on four quantum wells and uses a two phonon resonance depopulation scheme [17,18]. The basic segment consists of eleven well-barrier pairs and is repeated 50 times. The layer sequence, starting from the injection barrier, is as follows: 3.8, 1.2, 1.3, 4.3, 1.3, 3.8, 1.4, 3.6, 2.2, 2.8, 1.7, 2.5, 1.8, 2.2, 1.9, 2.1, 2.1, 2.0, 2.1, 1.8, 2.7, 1.8 nm [19]. The In_0_._36_Al_0_._64_As layers are denoted in blue bold font, and the In_0_._67_Ga_0_._33_As layers are denoted in normal font. The underlined layers (In_0_._36_Al_0_._64_As) are doped with silicon to a concentration of 4.0 × 10^17^ cm^−^^3^. The active region was sandwiched between two 500 nm thick lattice matched In_0_._53_Ga_0_._47_As layers, which were n-doped to 4.0 × 10^16^ cm^−^^3^. These layers were placed in order to increase the confinement factor of the waveguide. The structure was grown by solid-source Molecular Beam Epitaxy (MBE) on a Riber Compact 21T reactor, on a low doped InP (2.0 × 10^17^ cm^−^^3^) substrate, serving as the lower waveguide. The upper waveguide was formed by 1.5 μm thick InP (n = 3.0 × 10^16^ cm^−^^3^) followed by 1.5 μm thick InP (n = 1.0 × 10^17^ cm^−^^3^). The whole structure was capped with a heavily doped (2.0 × 10^18^ cm^−^^3^), 500 nm thick In_0_._53_Ga_0_._47_As contact layer. The upper waveguide and contact layer were grown by MOVPE. Epitaxial overgrowth was performed using AIXTRON 3 × 2” CCS MOVPE system [20].

**Figure 1:**
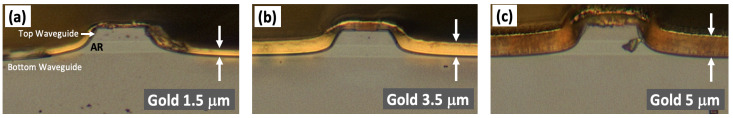
Optical microscope images of investigated RW AlInAs/InGaAs/InP QCLs with different electroplated gold thickness: (**a**) 1.5 μm, (**b**) 3.5 μm and (**c**) 5.0 μm.

Investigated QCLs were fabricated in RW geometry using standard processing technology, that is, a wet etching of mesa and Si_3_N_4_ layer deposition for electrical insulation [21] For the current injection, 15 μm wide contact windows were opened through the insulator layer using RIE-ICP plasma etching. The following types of contacts were used: Ti/Pt/Au ohmic contacts for low resistivity at the epi-side and AuGe/Ni/Au at the substrate side of the laser structure. In the next step, the contacts undergo thickening by electroplating. The devices with electroplated Au-metallization thicknesses of 1.5 μm, 3.5 μm and 5.0 μm were prepared to verify the influence of the electroplated gold thickness on the performance of the devices. Thicker gold layers were also deposited; however, above 5 μm the resulting quality of the gold layer degraded visually, indicating that a further increase of thickness would require additional development of the deposition process. The individual cleaved lasers, 2 mm long, were soldered epi-side up to Au-plated AlN submount, and then to copper mounts. Figure 1 shows optical microscope images of the ridge and top gold layer of exemplary devices with different gold thicknesses.

## 3. Results

The basic electro-optical and thermal characterization of devices with different electroplated gold layer thicknesses was performed. Additionally, micro-Raman spectroscopy was used in order to analyze strain introduced by a thick gold layer. All studied lasers were fabricated from the same epi-wafer in one processing run.

### 3.1. Electro-Optical Characterization

**Figure 2:**
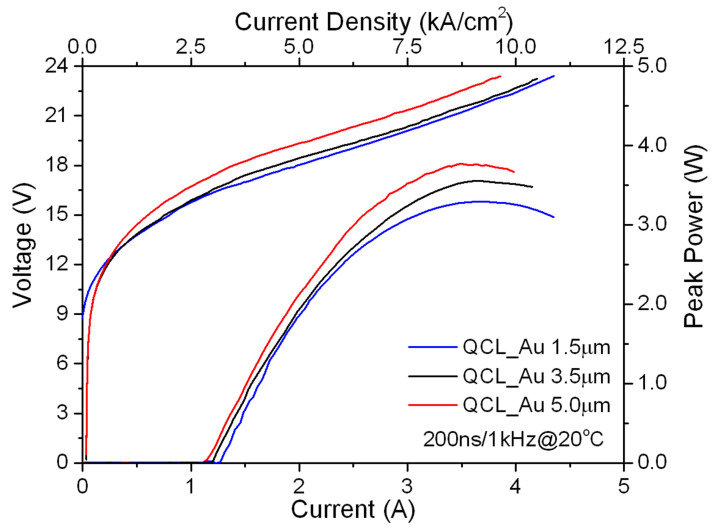
Room-temperature L-I-V characteristics for AlInAs/InGaAs/InP QCLs with different electroplated gold thickness.

Analysis of the impact of Au metallization thickness on QCLs parameters and the effectivity of heat dissipation was performed. First, the light-current-voltage (L-I-V) characteristics were measured for devices fabricated from the same epitaxial heterostructure, differing only in the gold layer thicknesses: 1.5 μm, 3.5 μm and 5.0 μm. Figure 2 presents L-I-V characteristics registered in pulsed mode at room temperature. The lasers were soldered epi-side up to an Au-plated AlN submount, and then to copper mounts. Lasers with uncoated facets were investigated. We used a typical set-up for QCL light–current–voltage characteristics measurement. The output power was measured using a TE (thermoelectric) cooled MCT (Mercury Cadmium Telluride) detector placed in front of the laser facet with anti-reflection-coated optics to improve light collection efficiency. 

The threshold current density decreases from 3.20 kAcm^−2^ for the laser with 1.5 μm gold layer thickness to 2.75 kAcm^−2^ for the laser with 5.0 μm gold layer thickness. At the same time, power at roll-over increases by 15%. The change of threshold current can be directly related to a decrease of the active region temperature and consequently the increase of laser gain [22]. However, the comparison of electro-optical parameters of QCLs, shown in Figure 2, shows only a slight improvement of these parameters for devices with thick metallization. This is related to the power supply conditions; short pulses and low pulse repetition rates (duty cycle dc = 0.02%) for which thermal problems are not yet critical. 

**Figure 3:**
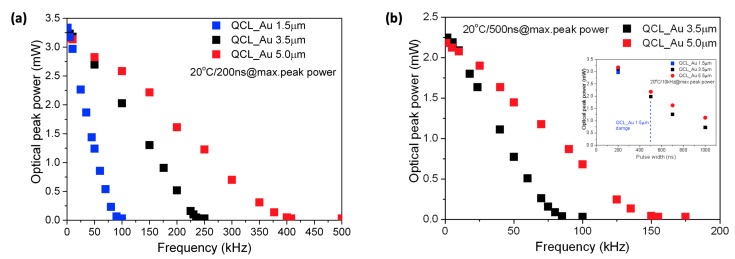
Optical peak power vs. frequency for investigated AlInAs/InGaAs/InP QCL with different gold thickness measured at following experimental conditions: (**a**) 20 °C/200 ns and (**b**) 20 °C/500 ns. Inset in (**b**) shows peak power vs. pulse length at constant frequency of 10 kHz.

To clearly see the improvement of heat dissipation efficiency with the thickest Au metallization thickness, we have measured the changes in the maximum emitted optical power as a function of the pulse repetition frequency for a constant pulse length and temperature. Figure 3a,b show how the output power decreases as the duty cycle is increased for constant pulse widths of 200 ns and 500 ns, respectively. In this case, the increase of the pulse repetition frequency introduces a greater thermal load for the devices. Under such operating conditions, the improvement in heat dissipation from the active region for QCLs with thick gold metallization is more pronounced. The heat generated during the pulse is much more efficiently dissipated for the device with the thicker top gold layer. This effect results from specific dynamic thermal properties of the QCL device structure, that is, the inter-pulse heating occurs on a much longer time scale than the intra-pulse thermal dynamics [23]. In this case, we observe the typical heat accumulation effect, which becomes more pronounced, as the thermal load increases and eventually leads to the thermal quenching of lasing operation.

From Figure 3a,b, it can be seen that for a 200 ns pulse width, the device with thick gold operates at much higher pulse repetition frequencies, reaching 500 kHz, as opposed to the thin gold device, which ceases to lase at 100 kHz. At longer pulses of 500 ns, the thin gold device was not able to operate at all, facing catastrophic damage before reaching the threshold.

**Figure 4:**
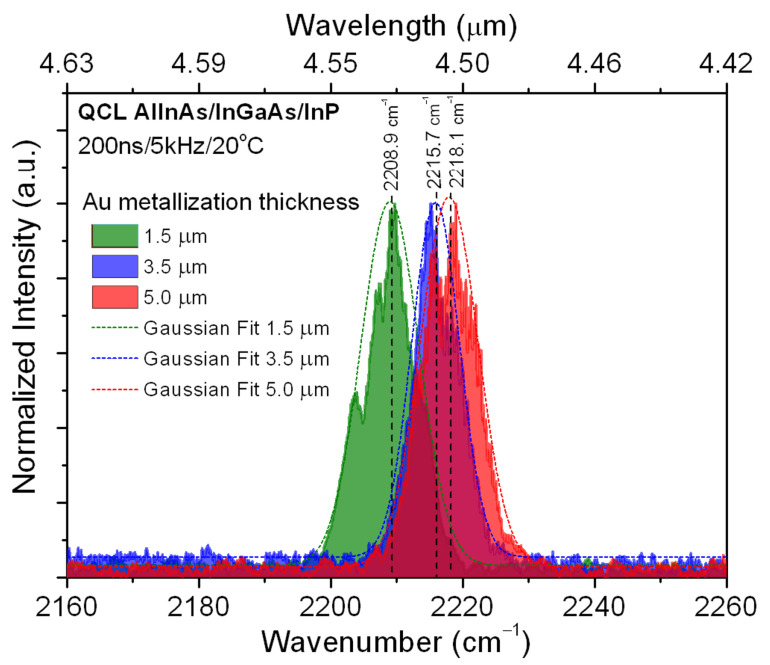
Emission spectra for AlInAs/InGaAs/InP QCLs with various gold metallization thickness: 5.0 μm (red line), 3.5 μm (blue line) and 1.5 μm (green line).

The effect of gold layer thickness on the temperature of the active region was further confirmed by measuring the emission spectra of the lasers. The devices were mounted on a thermoelectric cooler held at 20 °C. The laser emission was collected from the output facet and was sent to FTIR (Fourier Transform Infrared Spectroscopy) spectrometer. Spectra were registered with 0.125 cm^−1^ resolution. Figure 4 shows spectral characteristics registered for QCLs operated with 200 ns pulses and a 5 kHz repetition rate at a current of 1.25 A. The data present the red shift of emission wavelength with the decrease of the gold metallization thickness, which indicates that the temperature of the active region increases for devices with thin metallization. The shift of the peak wavelength, when moving from a device with a 5.0 μm gold layer to a 1.5 μm device equal to Δλ = 18 nm (9.05 cm^−1^), was observed. Considering an experimentally determined emission tuning rate of 0.3 nm/K, the temperature difference, between respective devices, can be estimated as ΔΤ = 60 K. 

### 3.2. Thermal Characterization

**Figure 5:**
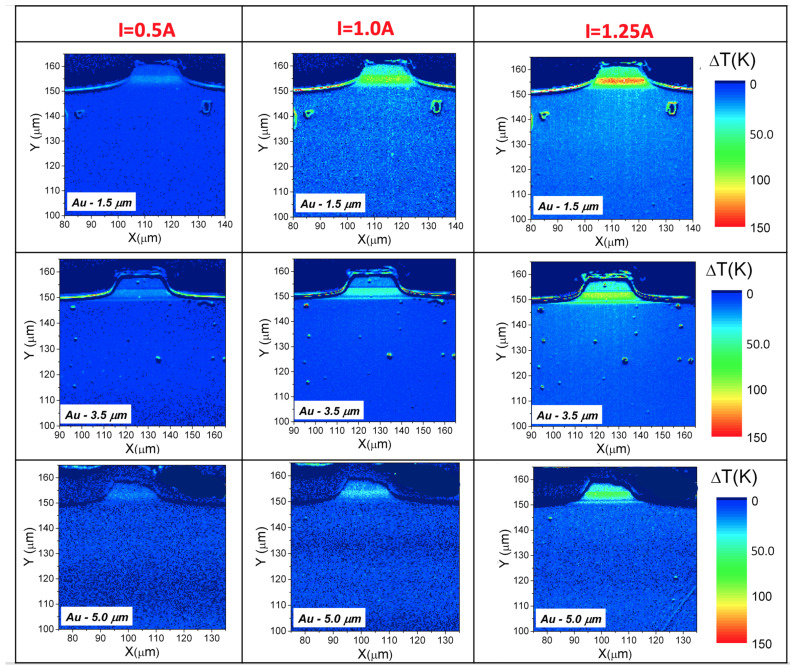
Thermal images of the ridge waveguide, epi-up mounted QCLs with different thickness of gold metallization, measured for pulse width of 10 μs, frequency 20 kHz and driving currents I = 0.5 A, 1.0 A and 1.25 A.

Direct information about the temperature of the device during pulsed operation can be obtained using the charge-coupled device (CCD)-based thermoreflectance (CCD-TR) [24,25], which allows for both high spatial (0.6 μm) and temperature resolution (below 1 K) imaging of temperature distribution on the laser facet. The optical part of the CCD–TR setup is based on the optical microscope equipped with a 100× long working distance objective. The sample is illuminated with a high intensity light emitting diode (LED) emitting at 630 nm. CCD-TR data allow for the localization of main heat sources and provide information on the efficiency of heat dissipation at different driving conditions. The technique is fast and accurate and provides information otherwise difficult to obtain. Figure 5 shows temperature distribution maps on the facet of the investigated AlInAs/InGaAs/InP QCLs with different gold metallization thicknesses: 1.5 μm, 3.5 μm and 5.0 μm. For comparison, the maps are presented in the same temperature scale and show an area of size 65 × 60 μm of the laser ridge and the active region.

**Figure 6:**
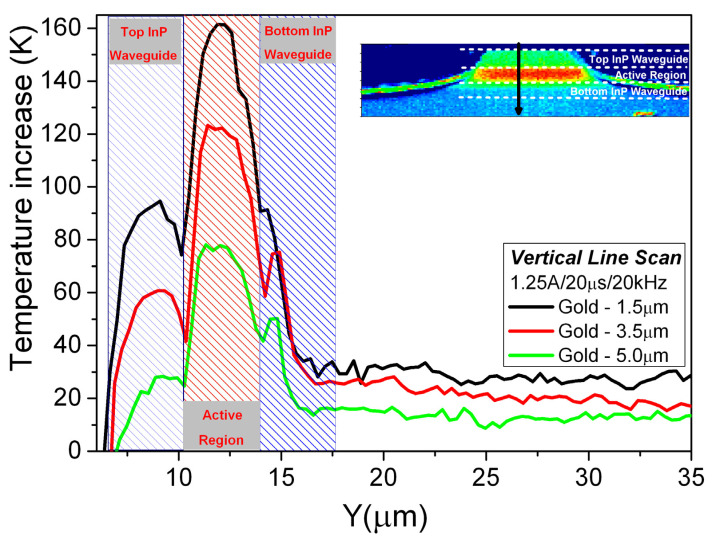
Vertical temperature profiles across the facet for devices with different gold thickness taken along the line as shown in the inset.

For all measured lasers, the maximum temperature increase was registered in the active area of the laser. Significant differences in the heat dissipation from the active area for lasers with different gold thicknesses can be observed. For lasers with thick Au metallization, the effect of gold thickness in spreading the heat generated at the active area is clearly visible, as the maximal temperature rise is lower and the heat distribution has a different shape. For the thick gold layer, heat is dissipated laterally and extracted through the metallization. In the case of a thin gold layer, more heat is dissipated in the direction of the heatsink, which is far less efficient. Above the active region (upper InP waveguide), the effect of heat accumulation is observed. To gain further insight into heat dissipation from the active area in the lasers with different gold thicknesses, temperature line scans across the facet were taken perpendicularly to the active layer as shown in Figure 6.

Besides the general effect of lowering device temperature with an increase of electroplated gold layer thickness, one can observe improved heat extraction from the upper waveguide, which clearly demonstrates the usefulness of thick gold in device heat management. 

**Figure 7:**
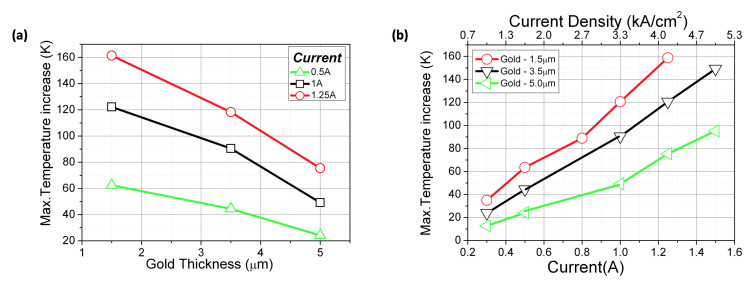
Laser core temperature as a function of gold thickness (**a**) and drive current (**b**).

The laser core temperature as a function of gold thickness and as a function of supply current is shown in Figure 7a,b. For a driving current of 1.25 A, an increase of gold layer thickness from 1.5 μm to 5.0 μm results in a 50% reduction of active region temperature. This is roughly the same value as in the case of a buried heterostructure (BH) mounted epi-down design, which is considered the most effective in terms of heat extraction [25]. However, considering that BH technology requires two-step epitaxy and complicated processing, thick electroplated gold technology seems to be a promising alternative, in particular in the case of cost-effective production technology. 

### 3.3. Micro-Raman Spectroscopy Characterization

Micro-Raman spectroscopy is a powerful tool for the investigation of processing-induced strain in semiconductor devices created by soldering a laser chip to the heatsink, packaging or metallization processes. The Raman spectrum parameters, such as intensity, width and peak frequency, provide information on the crystal quality, lattice strain and doping concentration [26]. The micro-Raman spectroscopy allows detailed studies with a spatial resolution in the sub-micron range. The measurements were performed at room temperature using an exciting Ar^+^ ion laser light of 488 nm. The light beam was guided into a confocal microscope with a 100× objective (numerical aperture = 0.9), providing a light spot diameter of 1 µm. The system was equipped with a monochromator with a focal length of 0.752 m and a nitrogen cooled CCD camera (1024 *×* 256 pixels). The spectral resolution of the Raman system was 0.625 cm^−^^1^ per CCD-pixel for an 1800 mm^−^^1^ grating. The Rayleigh elastic scattering was rejected by an edge filter. The motorized stage allowed the positioning of the light spot at different points on the sample surface with 0.1 µm steps in x–y directions. The Raman spectra were measured in the backscattering configuration with incident light perpendicular to the sample surface. The power of light was set below 1 mW to avoid laser induced surface heating. 

**Figure 8:**
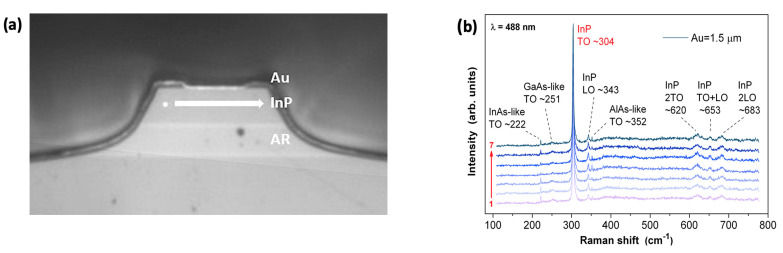
The image of the QCL facet showing position where Raman spectra were registered. The light spot (marked by white dot) was shifted along the InP cladding layer allowing measurements of Raman spectra at 7 different positions (**a**). Raman spectra registered at 7 different positions along the ridge (**b**).

The Raman spectra were measured on three lasers with different thicknesses of the gold layer. For each laser, the InP cladding layer was measured at seven different positions, with approximately 2.5 µm distance between points, along the ridge. The position in which the measurements were taken was in close proximity with contact metallization, as schematically shown in Figure 8a. The Raman spectra taken for the laser with a 1.5 micrometer thick gold layer are shown in Figure 8b. Spectra present the characteristic modes of the InP crystal. Although, for backscattering geometry on the (100) InP face, selection rules predict only the longitudinal optical LO mode at 343 cm^−^^1^. The transverse optical TO mode at 304 cm^−^^1^ is visible, which can be attributed to the failure of true backscattering geometry [26]. In the second-order optical range, the overtone bands are present at 620, 653 and 683 cm^−^^1^, and can be ascribed to 2 × TO, LO + TO, and 2 × LO modes, respectively. The dominant and sharp profiles of the InP TO peaks characterize the good crystal quality of the investigated layers.

**Figure 9:**
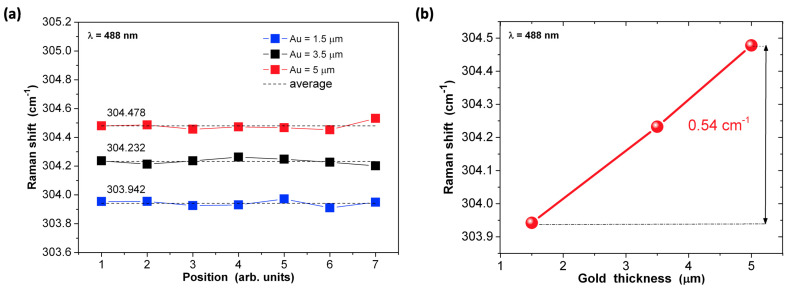
The frequencies of the InP TO modes in function of light spot positions obtained on the basis of Lorentzian profiles calculations. Numbers above each line indicate the average value of the InP TO peak frequency for each laser (**a**). Shift of InP TO frequencies in the function of gold layer thickness (**b**).

All spectra obtained for different light spot positions (see Figure 8b) show good reproducibility; the same frequencies of the main InP TO and LO modes, as well as other peaks which prove the existence of uniform stress in the cladding layer along the gold contact. The same is true for lasers with thicker gold layers (3.5 µm and 5.0 µm), although peak positions are shifted to slightly higher frequencies. The spectra were normalized to the maximum intensity value of the InP TO modes for each laser. Figure 9a shows the frequencies of the InP TO modes as a function of light spot positions obtained by the fitting to Lorentzian profiles. The difference in InP TO mode frequencies exists, as shown in Figure 9b. For thicker gold layers, the InP TO peaks shift toward higher frequencies, which can be attributed to the mechanical stress existing in InP cladding layers.

Table 1 lists the shifts of average InP TO phonon line frequency in QCLs with different thicknesses of electroplated gold layer with respect to the InP TO phonon line of the substrate, which is treated as a reference line (304.0 cm^−1^ [26]). The values of stress in the InP cladding close to gold metallization were calculated using the following relation between Raman shift and stress [27]:

∆ω = −2 × 10^−9^ σ

Here, ∆ω is given in units of cm^−1^ and σ in Pascal (Pa). Calculated stress values are shown in Table 1.

The above results show that gold layers electroplated on top of laser ohmic contact, even in the case of 5.0 µm thickness, introduce only moderate compressive strain in the top InP cladding. These values are well below the critical value (~34 MPa @RT) for the generation of misfit dislocation, causing plastic relaxation in the cladding and active layers and eventually leading to device failure [19,28,29].

## 4. Conclusions

In this paper, we have examined the influence of electroplated gold thickness on the thermal and electro-optical properties of mid-IR AlInAs/InGaAs, InP QCLs. The investigated QCLs were fabricated in RW geometry using standard processing technology. The devices with electroplated Au-metallization thicknesses of 1.5 μm, 3.5 μm and 5.0 μm were prepared. The as-cleaved lasers, 2 mm long, were soldered epi-side up to Au-plated AlN submount, and then to copper mounts.

Experimental results show a significant reduction of the temperature of the QCL active region with increasing gold layer thickness. For QCLs with a 5.0 μm gold thickness, we observed a 50% reduction of the active region temperature. An improvement of key electro-optical parameters, that is, a reduction of threshold current density and an increase of maximum emitted power for structures with thick gold was observed. The effect of gold layer thickness on the temperature of the active region was further confirmed by measuring the emission spectra of the lasers. The shift of the peak wavelength when moving from a device with a 5.0 μm gold layer to a 1.5 μm device equal to Δλ = 18 nm (9.05 cm^−1^) was observed. Considering an experimentally determined emission tuning rate of 0.3 nm/K, the temperature difference between respective devices can be estimated as ΔΤ = 60 K. 

Direct information about the temperature of the device during pulsed operation was obtained using the CCD- thermoreflectance. Significant differences in the heat dissipation from the active area for lasers with different gold thicknesses were observed. For a thick gold layer, heat is dissipated laterally and extracted through the metallization. In the case of a thin gold layer, more heat is dissipated in the direction of the heatsink, which is far less efficient. Above the active region (upper InP waveguide), the effect of heat accumulation is observed.

The Raman spectra were measured with micrometer spatial resolution at different positions across the ridge in close proximity to contact metallization. All spectra obtained for a given gold layer thickness showed the same frequencies of the main InP TO and LO modes, which proves the existence of uniform stress in the cladding layer along the gold contact. With increasing gold thickness, the InP TO peaks shift toward higher frequencies. For the thickest, 5.0 μm gold layer, the compressive strain introduced by metallization was estimated as 7.25 MPa, which is well below the critical value for the creation of misfit dislocations. 

It has been demonstrated that a thick electroplated Au top contact in ridge waveguide geometry adds a shorter path for heat extraction and greatly improves heat removal from the active region, which makes this technology very attractive from the point of view of production technology.

## Figures and Tables

**Table 1:** Raman shift of the TO InP phonon line and calculated stress values in reference to InP TO phonon mode of the substrate (ω = 304.0 cm^−1^ [26]). Positive σ value means tensile stress, while negative value indicates existing of compressive stress.

Sample	Δω [cm^−1^]	σ [MPa]
QCLs with 1.5 Au	−0.058	0.88
QCLs with 3.5 Au	0.232	−3.5
QCLs with 5.0 Au	0.478	−7.25

## Data Availability

The data that support the findings of this study are available from the corresponding author upon reasonable request.
